# Promotion of Overall Water Splitting Activity Over a Wide pH Range by Interfacial Electrical Effects of Metallic NiCo‐nitrides Nanoparticle/NiCo_2_O_4_ Nanoflake/graphite Fibers

**DOI:** 10.1002/advs.201801829

**Published:** 2019-01-15

**Authors:** Zhihe Liu, Hua Tan, Daobin Liu, Xiaobiao Liu, Jianping Xin, Junfeng Xie, Mingwen Zhao, Li Song, Liming Dai, Hong Liu

**Affiliations:** ^1^ Institute for Advanced Interdisciplinary Research (IAIR) University of Jinan Shandong 250022 China; ^2^ State Key Laboratory of Crystal Materials Shandong University Jinan Shandong 250100 China; ^3^ State Key Laboratory of Crystal Materials Shandong University Jinan Shandong 250100 China; ^4^ National Synchrotron Radiation Laboratory CAS Hefei Science Center University of Science and Technology of China Hefei Anhui 230026 P. R. China; ^5^ School of Physics and Microelectronics Shandong University Jinan Shandong 250100 China; ^6^ College of Chemistry Chemical Engineering and Materials Science Shandong Normal University Jinan 250014 China; ^7^ Center of Advanced Science and Engineering for Carbon (Case4carbon) Department of Macromolecular Science and Engineering Case Western Reserve University 10900 Euclid Avenue Cleveland OH 44106 USA

**Keywords:** electrocatalysts, interfacial electrical effects, overall water splitting

## Abstract

Many efforts have been made to develop bifunctional electrocatalysts to facilitate overall water splitting. Here, a fibrous bifunctional 3D electrocatalyst is reported for both the hydrogen evolution reaction (HER) and the oxygen evolution reaction (OER) with high performance. The remarkable electrochemical performance is attributed of the catalysts to a number of factors: the metallic character of the three components (i.e., Ni_3_N, CoN, and NiCo_2_O_4_); the electronic structure, nanoflake‐nanosphere network with abundant electroactive sites, and the electric field effect at the interfaces between different components. The oxide–nitride/graphite fibers have the lowest overpotential requirements of 71 and 183 mV at 10 mA cm^−2^ for HER and OER in alkaline medium, respectively. These values are comparable to those of commercial Pt/C (20 wt%) and RuO_2_. The electrodes also show a response to HER and OER in both neutral and acid media. Furthermore, the 3D structure can be highlighted by all‐round electrodes for overall water splitting. The calculations on the changes in electrons transfer and the Femi level from oxides to oxides/nitrides reveal that the observed superb electrocatalytic performance can be attributed to the presence of Ni_3_N and CoN derived from the in situ nitridation of NiCo_2_O_4_.

Electrocatalytic water splitting is regarded as a promising approach to a sustainable, secure, and eco‐friendly source of hydrogen‐fuel energy.^1^ The water splitting reaction can be classified into two half reactions occurring at electrodes, namely the oxygen evolution reaction (OER) and the hydrogen evolution reaction (HER).^2^ The efficiencies of both half reactions are key factors determining overall water splitting performance. Due to various thermodynamic and kinetic hindrances, however, a large overpotential inhibits practical applications of these catalysts.^3^ There is a need to reduce losses in electrocatalysts to lower the applied voltage required for OER and HER.^4^ Previously, considerable progress has been made towards efficient HER and OER catalysts with earth‐abundant materials, such as transition metal carbides, sulfides, phosphides, selenides, nitrides owing to their low cost, abundance, and high efficiency.^5^ Nevertheless, many strategies for improving electrochemical reactions have been restricted to monofunctional electrocatalysts owing to the reverse redox processes occurring at the anode and cathode. Some electrocatalysts have been fabricated by combining HER and OER active components to achieve bifunctional electrocatalytic performance.^6^ Although some progress has been made, it remains difficult to coordinate electrode reactions for overall water splitting in an integrated electrolyzer for practical use over wide pH ranges.^7^ Bifunctional electrocatalysts would be desirable, with high catalytic performance towards both HER and OER in the same electrolyte in broad pH region.

Recently, rational improvements have been made by increasing the number of active sites through nanostructuring of catalysts, dispersing the catalysts on supports with high surface areas, and developing shape‐controlled catalysts with specific crystalline faces.^8^ Furthermore, some improvements have been made by increasing intrinsic activities, through alloying and doping of the catalysts.^9^ Hybrid electrocatalysts show great promise for high‐performance HER and OER. However, there have been few reports on the use of hybrid structures based on metallic components to boost the electrochemical performance. Xue et al. reported a Mott–Schottky electrocatalyst consisting of Co/CoP for overall water splitting.^10^ Compared with Mott–Schottky structures, metallic interfaces transport electrons more efficiently owing to interfacial electric field effects and possible synergetic effects between the different parts of the hybrid material and enhanced interfacial electron transfer efficiency between the different metallic components. Metal nitrides show superior metallic characteristics owing to their distinct electronic structure and properties, such as low electrical resistance and corrosion resistance.[[qv: 5d,11]] The introduction of N atoms has been confirmed to strongly affect the electronic structure of the metal nanocomposites through charge‐transfer or concomitant structural modification.^12^ Metal oxide formed on the surface of metal nitrides during initial electrochemical processes could act as active components to readily absorb OH^−^ to facilitate HER and OER while the metal nitride core, compared to metal oxides, can expedite electron transport from the conductive support to the catalyst surface.^13^ Lou and co‐workers reported on NiCo_2_O_4_ hollow spheres with metallic properties, which exhibited high capacities as supercapacitors, indicating that NiCo_2_O_4_ has a spinel structure and possess good conductivity.^14^ Therefore, it is a good option to develop hybrid structures based on metallic components as bifunctional electrocatalysts.

In this regard, 3D materials with porous structures have been widely used as electrode materials. The compact pore structure gives the materials high surface areas, which expands the contact area between the electrode and electrolyte compared with that of bulk solid structures.[[qv: 11b]] This structure further facilitates electron transport from the catalyst to the substrate and promotes the release of the evolved gas bubbles to maximize the catalytic performance.^15^ Moreover, electrochemical tests require the catalysts and conductive substrate to be linked, often through the use of polymer binder as a film‐forming agent.^16^ However, this modification increases the series resistance and blocks active sites, reducing the activity. Thus, it is promising to synthesize 3D hybrid structures grown on highly conductive substrates as bifunctional electrocatalysts to enhancing the efficiency of both HER and OER.

Herein, we develop a facile method to fabricate 3D NiCo‐nitrides nanoparticles/NiCo_2_O_4_ nanoflakes/graphite fibers (denoted as NiCo‐nitrides/NiCo_2_O_4_/GF) by assembling nitride–oxides hetero‐nanostructures onto graphite fibers. We used a simple electrochemical deposition, followed by in situ nitridation. The resultant NiCo‐nitrides/NiCo_2_O_4_/GF can act as bifunctional electrocatalysts for HER and OER over a broad pH range. The graphite fibers were more desired alternative compared with other conductive substrates due to their more conductive nature associated with the higher degree of graphitization, as evidenced by Raman spectrum (Figure S1, Supporting Information). Our results revealed synergistic effects between the metallic NiCo‐nitrides nanoparticles and NiCo_2_O_4_ nanoflake hybrid structures. The electric field at the interface of the NiCo‐nitrides/NiCo_2_O_4_ formed on the graphite fibers provides a pathway for electron transfer. Furthermore, NiCo‐nitrides offer more exposed active sites for electrocatalytic water splitting. As a result, our NiCo‐nitrides/NiCo_2_O_4_/GF shows excellent electrocatalytic activity and stability towards overall water splitting over a wide range of pH values. The newly developed 3D structure could provide a large surface area to act all‐round electrode for overall water splitting.

As shown in **Scheme** **1**, a layer of mixed nickel–cobalt hydroxides was assembled on the surface of a bundle of graphite fibers by a simple co‐electrodeposition method in a solution of cobalt and nickel nitrate. The graphite fibers acted as both the electrode and substrate. The nickel–cobalt hydroxides on the graphite fibers were in situ transformed to NiCo_2_O_4_ by a pyrolysis process (denoted as NiCo_2_O_4_/GF). Finally, NiCo_2_O_4_ was partially converted into NiCo‐nitrides by thermal ammonolysis, to form hetero‐nanostructured NiCo‐nitrides/NiCo_2_O_4_/graphite fibers (denoted as NiCo‐nitrides/NiCo_2_O_4_/GF) (see Figures S3 and S5 in the Supporting Information for more details).

**Scheme 1:**
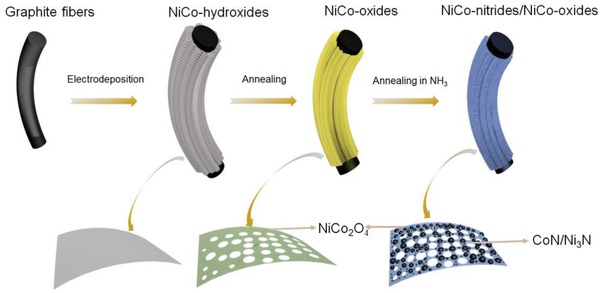
Schematics of the fabrication processes of NiCo‐nitride/NiCo_2_O_4_ supported graphite fibers.

The graphite fiber is a continuous filament with a high graphitization degree and 7–9 µm in diameter (inset of **Figure** **1**a). After electrodeposition and pyrolysis, a layer of uniformly distributed interconnected nanoflakes formed on the graphite fibers (Figure 1a). The thickness of the nanoflakes on the graphite fibers was ≈500 nm (Figure S6, Supporting Information). High‐magnification images of the samples showed that nanoflakes with a lateral size of ≈10 nm were interconnected and stood out vertically from the surface of the graphite fibers (Figure 1b). Moreover, the nanoflakes possessed a uniform distribution of pores connected at their edges to form a 3D network structure. The many edges of the nanoflakes and the walls of the pore gave the nanostructures a large surface area and many electroactive sites for redox reactions. A transmission electron microscope (TEM) image of the sample recorded by Figure 1b confirmed the porous structure and we clearly observed lattice fringes in the high‐resolution TEM (HRTEM) image (Figure S7, Supporting Information). The image showed planes with a lattice spacing of 0.245 nm corresponding to the (311) plane of NiCo_2_O_4_ (Figure S8, Supporting Information). Although the interconnected nanostructures remained tightly attached to the surface of the graphite fibers after nitridation, the morphology of nanostructures was altered from that of the non‐nitrided sample. A large number of nanospheres with diameters in the range of 30–50 nm assembled on the surface of the interconnected nanoflakes to form a unique 3D beaded fabric‐like nanostructure. This structure offers a pathway for rapid electron‐transport from the interface of the electrodes and electrolyte to the intrinsic electrically conductive graphite fibers (Figure 1c,d). The TEM images in Figure 1e, further revealed that the nanoflakes with a porous structure were ultrathin and connected with the spheres, as observed in our SEM results. The HRTEM image shown in Figure 1f was acquired from the single nanosphere indicated by the red square in Figure 1e and confirmed the polycrystalline nature of the nanospheres. Lattice fringes were clearly observed with three planes, having lattice spacings of 0.203 and 0.248 nm, respectively, corresponding to the (111) plane of N_3_N, the (111) plane of CoN. Lattice fringes with lattice spacings of 0.245 and 0.469 nm can be indexed to the (311) and (111) plane of NiCo_2_O_4_, respectively. We observed clear interfaces between NiCo_2_O_4_ and CoN or Ni_3_N. TEM mapping images of NiCo‐nitrides/NiCo_2_O_4_ in Figure 1g show the distribution of N, O, Ni, and Co, indicating that the nanoflakes were coated with the nanoparticle structures. Both the nanoflakes and nanoparticles were composed of Ni and Co; however, N was only present in the nanoparticles and O was only present in the nanoflakes, which corresponded with our HRTEM and SEM results.

**Figure 1:**
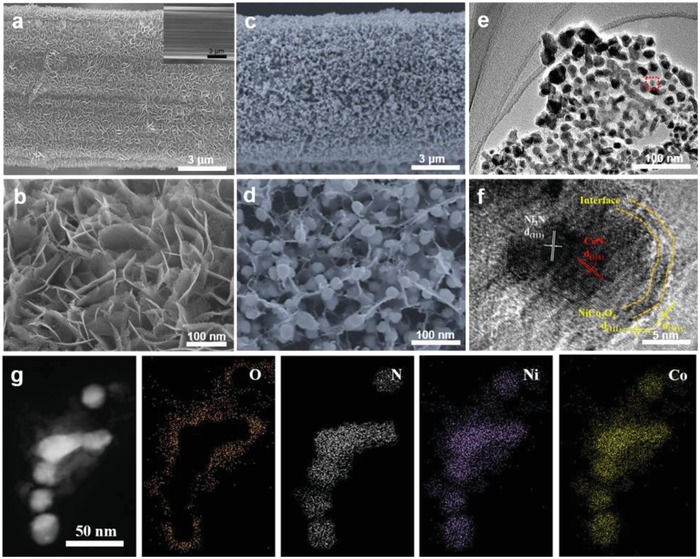
Structural and compositional characterization. a,b) SEM images of NiCo_2_O_4_/GF. Inset: bare graphite fiber; c,d) SEM images of NiCo‐nitrides/NiCo_2_O_4_/GF; e) TEM images of the obtained NiCo‐nitrides/NiCo_2_O_4_; f) HR‐TEM image of NiCo‐nitrides/NiCo_2_O_4_. g) TEM elemental mapping images of NiCo‐nitrides/NiCo_2_O_4_ showing the distributions of Ni, Co, O, and N.

We then carried out X‐ray diffraction (XRD) measurements to identify the phase of the samples. As shown in **Figure** **2**a, the main peak of NiCo_2_O_4_/GF at 36.69° can be indexed to the cubic‐structured NiCo_2_O_4_.^14^ After nitridation, the added peaks located at 36.2° and 44.8° correspond to the cubic‐structured CoN and hexagonal‐structured Ni_3_N, respectively.[[qv: 11a,17]] X‐ray photoelectron spectroscopy analyses were also used to probe the chemical states of the NiCo_2_O_4_ and NiCo‐nitrides/NiCo_2_O_4_. According to the X‐ray photoelectron spectrometer (XPS) analysis, the survey spectra of both the NiCo_2_O_4_ supported on graphite fibers demonstrate that the Ni, Co, O, C elements are the main components in NiCo_2_O_4_. After nitridation, the intensity of O1s decreases, the peak of N1s appears, which further confirms the partial transformation from NiCo_2_O_4_ to NiCo‐nitrides (Figure 2b). According to the Ni2p spectra of NiCo_2_O_4_, the Ni2p spectrum in Figure 2c was composed of two spin–orbit doublet characteristics and two Sat, the peaks at ≈873.9 and 856.5 eV was fitted with Ni^3+^, whereas those at 872.3 and 855.2 eV corresponding to Ni^2+^, which was consistent with NiCo_2_O_4_ reported.^18^ After nitridation, the added Ni^+^ appeared in the Ni2p spectrum in Figure 2c of NiCo‐nitrides/NiCo_2_O_4_/GF as well as Ni^2+^ and Ni^3+^, which indicates the Ni^2+^ and Ni^3+^ were reduced to metallic state in NH_3_ atmosphere due to the reduction of NH_3_.^19^ The Ni^+^ resulted from the multi high‐ and low‐mixed valence. Correspondingly, the Co2p emission spectrum was composed of two spin–orbit doublet characteristics and two satellites (indicated as “Sat”). The peaks of NiCo_2_O_4_ around 796.5 and 780.4 eV were well indexed to Co^3+^, whereas those at 798.1 and 782.3 eV related to Co^2+^ (Figure 2d). The content of Co^2+^ was increased based on the decreased content of Co^3+^ in the Co2p spectrum of the nitridated NiCo_2_O_4_ referring to the two spin–orbit doublets, characteristic of Co^2+^ and Co^3+^ in Figure 2d. Both the Ni_2p_ and Co_2p_ negatively shift towards to low bonding energy implying the low valence‐state of Ni and Co forming after nitrogenization. Moreover, for N1s spectrum in Figure S9 (Supporting Information) the characteristic peak was located at around 399 eV, which can be assigned to the nitrogen in a metal nitride.^20^

**Figure 2:**
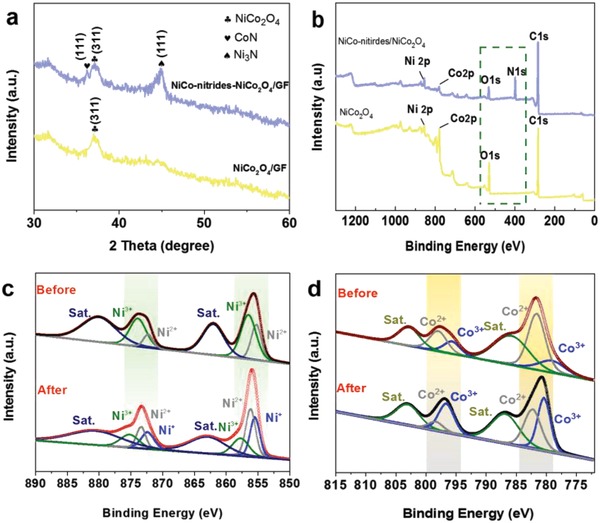
a) XRD patterns of NiCo_2_O_4_ and NiCo‐nitrides/NiCo_2_O_4_; b) XPS full survey spectra of NiCo_2_O_4_/GF and NiCo‐nitrides/NiCo_2_O_4_/GF; c) Ni 2p of NiCo_2_O_4_ before and after nitridation; d) Co 2p of NiCo_2_O_4_ before and after nitridation.

To verify the components of the NiCo‐nitrides/NiCo_2_O_4_/GF, ex situ X‐ray absorption fine structure (XAFS) spectroscopy has been carried out. The Fourier transformed extended XAFS (FT‐EXAFS) data for NiCo‐nitrides/NiCo_2_O_4_/GF and NiCo_2_O_4_/GF are shown in **Figure** **3**a–d. The X‐ray absorption near‐edge spectra (XANES) of the NiCo‐nitrides/NiCo_2_O_4_/GF showed a tendency for the pre‐edge feature to shift to lower energy bonding compared with that of NiCo_2_O_4_/GF (Figure 3a). We attribute this result to nitridation reducing the valence of the metal, as observed in our XPS results. Moreover, the FT‐EXAFS characteristic features located at 1.37 and 2.25 Å, corresponded to Ni–N/O and Ni–Ni correlations.[[qv: 12a]] In the FT‐EXAFS spectra, we noted a sharp increase of the FT intensity of the Ni–Ni bonding in the sample after nitridation. The strengthened Ni–Ni bonding attributed to the nickel nitrides and cobalt nitrides forming during the nitridation because of the intensity of the metal nitride features with typical metallic characteristics being higher than those of metal oxides.^21^ Similarly, we confirmed that Co was reduced to a low valence state by nitridation to form cobalt nitrides. All of the above ex situ results demonstrated that the metallicity of the sample was improved after nitridation.

**Figure 3:**
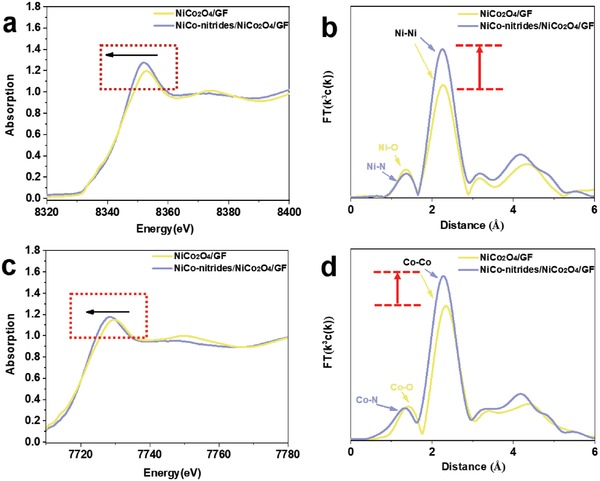
In‐situ XAS characterization of NiCo‐nitride/NiCo_2_O_4_/GF and NiCo_2_O_4_/GF a,b) XANES profiles for Ni K‐edge and corresponding FT‐EXAFS spectra of NiCo‐nitride/NiCo_2_O_4_/GF and NiCo_2_O_4_/GF. c,d) XANES profiles for Co K‐edge and corresponding FT‐EXAFS spectra of NiCo‐nitride/NiCo_2_O_4_/GF and NiCo_2_O_4_/GF.

The electrocatalytic HER activities of NiCo_2_O_4_/GF and NiCo‐nitrides/NiCo_2_O_4_/GF electrodes were evaluated directly as the working electrodes in N_2_‐saturated 1.0 m KOH electrolyte using a standard three‐electrode system. For comparison, similar measurements for bare graphite fibers and commercial Pt/C (20 wt%) loaded on a glassy carbon electrode were also performed. Linear sweep voltammetry curves in **Figure** **4**a shows that NiCo‐nitrides/NiCo_2_O_4_/GF exhibits the highest HER activity with the lowest overpotentials (η) of 71 and 180 mV to obtain a current density of 10 and 50 mA cm^−2^, respectively, compared with NiCo_2_O_4_/GF electrode and graphite fibers, which is close to that of commercial Pt/C (20 wt%). The NiCo_2_O_4_/GF electrode featured an overpotential (η) of 307 mV, which is higher than that of the NiCo‐nitrides/NiCo_2_O_4_/GF. The bare graphite fibers without any active materials show a small response. The remarkable activity for HER of the NiCo‐nitrides/NiCo_2_O_4_ was further assessed by its inherently higher current density over the potential ranges measured. We evaluated the catalytic kinetics of the samples are evaluated from their Tafel plots (Figure 4b). The NiCo‐nitrides/NiCo_2_O_4_/GF reveals higher kinetics for HER with a low Tafel slope of 58 mV dec^−1^ compared with that of the NiCo_2_O_4_/GF (132 mV dec^−1^) and the results was also close to that of Pt/C (35 mV dec^−1^), indicating that the NiCo‐nitrides/NiCo_2_O_4_/GF shows promise as a HER electrocatalyst. Electrochemical impedance spectroscope (EIS) tests on NiCo_2_O_4_/GF and NiCo‐nitrides/NiCo_2_O_4_/GF were conducted at the same applied potential (−0.38 V vs RHE) to further investigate the kinetics with catalytic activity (Figure 4c). The NiCo‐nitrides/NiCo_2_O_4_/GF features smaller semicircular arc than that of the NiCo_2_O_4_/GF suggesting its fast electron transfer during the electrochemical reactions. The result corresponds to the low overpotentials associated with the NiCo_2_O_4_/GF and the NiCo‐nitrides/NiCo_2_O_4_/GF. The results of a stability test conducted for the NiCo‐nitrides/NiCo_2_O_4_/GF and the NiCo_2_O_4_/GF at a constant potential of −0.07 V (vs RHE) and −0.369 V (vs RHE), respectively, are shown in Figure S10 in the Supporting Information. Almost no decrease in current density occurred over 40 h for the NiCo‐nitrides/NiCo_2_O_4_/GF and the NiCo_2_O_4_/GF via continuous chronoamperometric response (*i–t*) under alkaline condition. The morphology of the NiCo‐nitrides/NiCo_2_O_4_/GF changed little (Figure S11, Supporting Information). These results confirmed that NiCo‐nitrides/NiCo_2_O_4_/GF featured remarkable HER catalytic activity with low overpotential, high current density, and low charge transfer resistance in addition to good chemical stability. The main electrochemical properties arose from the 3D bead‐curtain‐like nanostructures formed by the unique porous and ultrathin nanoflakes decorated with nitride nanospheres. This structure provides many accessible active sites for the adsorption of H_2_O and short diffusion pathways for charge transfer to a charge collector. The hybrid structures of NiCo_2_O_4_, Ni_3_N, and CoN play a vital role in contributing to the high performance.

**Figure 4:**
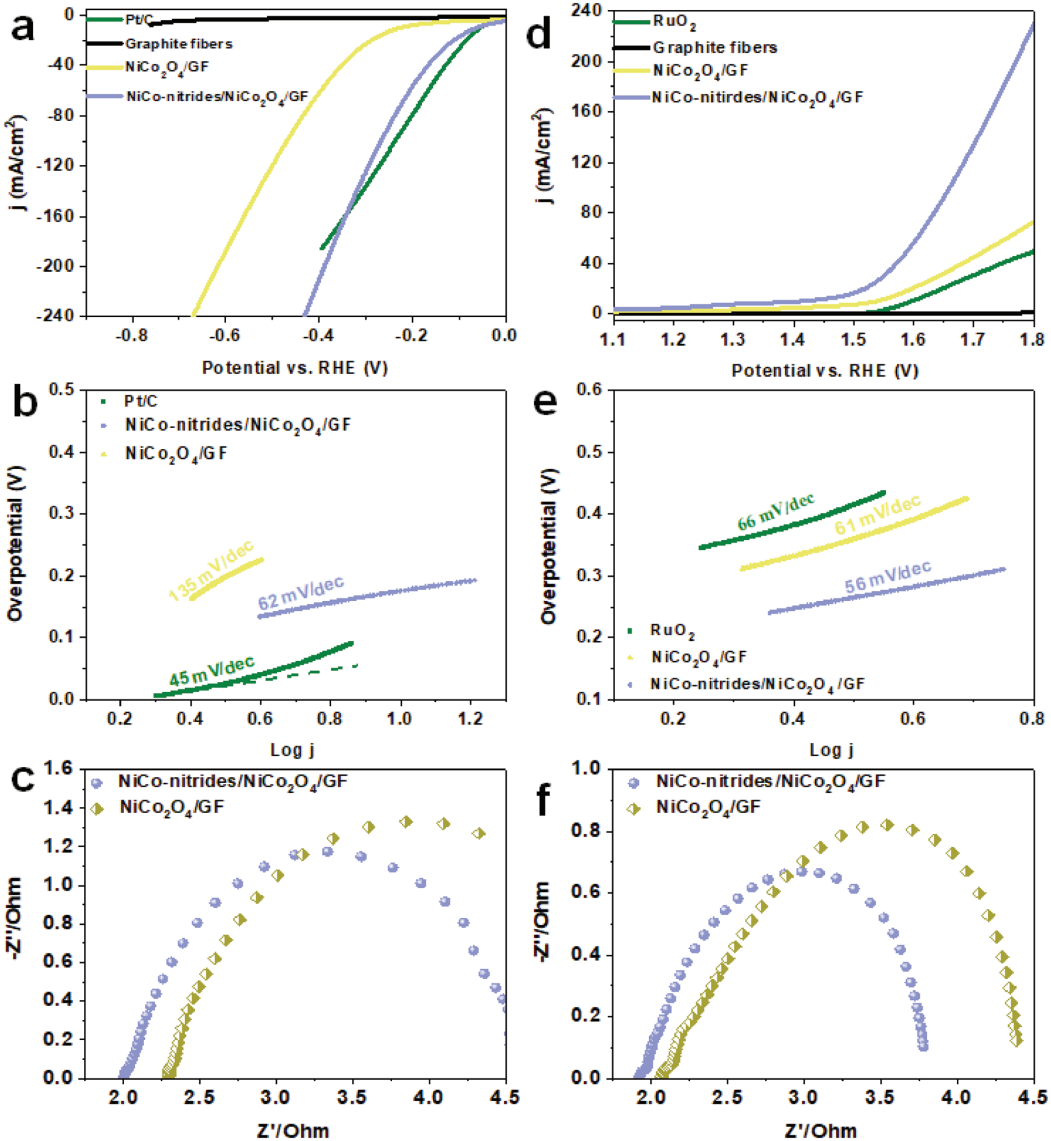
HER and OER activities of the catalysts in 1 m KOH a) linear sweep voltammetry (LSV) curves for HER without IR‐corrected; b) Corresponding Tafel plots of all samples in 1 m KOH; c) EIS Nyquist plots at −0.38 V; d) LSV curves for OER without IR‐correction; e) Corresponding Tafel plots; f) EIS at 1.53 V for different electrocatalysts.

An ideal bifunctional electrocatalyst should possess bifunctional activities toward HER and OER, which could simplify water splitting systems and lower total costs. We expected that NiCo_2_O_4_/GF and NiCo‐nitrides/NiCo_2_O_4_/GF electrodes should feature good OER activity owing to their graphite substrate and the metallic characteristics of the active nanostructures on the graphite fibers. The OER activities of NiCo_2_O_4_/GF and NiCo‐nitrides/NiCo_2_O_4_/GF electrodes were evaluated in O_2_‐saturated 1.0 m KOH with a scan rate of 5 mV s^−1^. According to the polarization curves in Figure 4d, the NiCo‐nitrides/NiCo_2_O_4_/GF exhibits the highest OER activity with the lowest overpotentials (η) of 183 and 344 mV to obtain current densities of 10 and 50 mA cm^−2^. By comparison, 300 and 366 mV overpotentials at a current density of 10 mA/cm^−2^ are required for NiCo_2_O_4_/GF and RuO_2_. Furthermore, the Tafel slope for NiCo‐nitrides/NiCo_2_O_4_/GF (56 mV dec^−1^) was the smallest among all the was the smallest among all the electrocatalysts tested including NiCo_2_O_4_/GF (61 mV dec^−1^) and RuO_2_ (66 mV dec^−1^) (Figure 4e). As shown in Figure 4f, the electrochemical impedance of the NiCo‐nitrides/NiCo_2_O_4_/GF is smaller than that of NiCo_2_O_4_/GF, further confirming the lower charge transfer resistance during the electrochemical reactions (For more details about the equivalent circuit of EIS see Figure S12 in the Supporting Information). The stability test of the NiCo‐nitrides/NiCo_2_O_4_/GF and the NiCo_2_O_4_/GF in Figure S13 (Supporting Information) was also tested by continuous chronoamperometric response (*i–t*) measurements under alkaline condition at the applied potential of 1.41 V (vs RHE) and 1.60 V (vs RHE), respectively. The results show almost negligible degradation during 40 h of continuous operation, which confirms the excellent durability. It is generally accepted that transition metal oxides on the surface of transition metal nitrides as electrocatalysts serves as actual catalytic sites during OER process.[[qv: 11b]] Notably, in our system, NiCo‐oxides can directly work as actual electroactive sites for OER. Compared to the generated oxides or hydroxides on the surface of transition metal nitrides, NiCo‐oxides based on the in situ formed NiCo‐nitrides/NiCo_2_O_4_ provide larger active area. The enhanced performance of NiCo‐nitrides/NiCo_2_O_4_/GF should be ascribed to the NiCo‐nitrides/NiCo_2_O_4_ structures with electric field effect to contribute to electron transfer and expanded electroactive area. A comparison list in Table S1 (Supporting Information) shows the HER and OER activities of previously reported non‐noble bifunctional electrocatalysts and the NiCo‐nitrides/NiCo_2_O_4_/GF synthesized in this work. These data illustrate that both the HER and OER activities of the NiCo‐nitrides/NiCo_2_O_4_/GF are better activity than that of most other non‐noble electrocatalysts reported to date.

According to the above studies, we clarified that the NiCo‐nitrides/NiCo_2_O_4_/GF acted as bifunctional electrocatalysts with high catalytic performance towards both HER and OER in alkaline media. The linear sweep voltammetry (LSV) curves of the NiCo‐nitrides/NiCo_2_O_4_ displayed overpotentials of 170 and 280 mV (vs RHE) at 20 mA cm^−2^ for HER and OER in a three‐electrode system, respectively, indicating that the overall potential window can be 1.68 V at 20 mA cm^−2^ in **Figure** **5**a. Furthermore, we applied the NiCo‐nitrides/NiCo_2_O_4_ electrodes to assemble a water splitting electrolyzer with a two‐electrode system, which was tested in 1 m KOH. The applied potential was larger than 1.5 V, and hydrogen and oxygen bubbles were sustainably released as the potential was increased (Figure 5b). The durability test was maintained at 10 mA cm^−2^ in 1 m KOH, which also showed little degradation after 40 h (Figure S14, Supporting Information). To confirm the improved performance of synthesized samples, we compared the electrochemically active surface area (EASA) of the NiCo‐nitrides/NiCo_2_O_4_/GF and the NiCo_2_O_4_/GF by measuring the electrochemical double‐layer capacitance (*C*_dl_). The NiCo‐nitrides/NiCo_2_O_4_/GF delivered a higher C_dl_ (114.4 mF cm^−2^) than that of NiCo_2_O_4_/GF (90.13 mF cm^−2^) and these values are both related to EASA (Figure S15, Supporting Information). Previous reports have proved that the activity increases owing to the greater electrochemical surface area.^22^ Our NiCo‐nitrides/NiCo_2_O_4_/GF possess a notably greater exposure of active sites. Furthermore, we could operate the electrolyzer with a single AAA battery of 1.5 V (Figure 5e). We noted that a difference in the increases of respective volumes of hydrogen and oxygen gases from the cathode and anode derived from the electrocatalytic overall water splitting of the NiCo‐nitrides/NiCo_2_O_4_/GF and NiCo_2_O_4_/GF. The volume–time curves for both hydrogen and oxygen were recorded by automatic online trace gas analysis system‐gas chromatography (Labsolar‐6A, Beijing Perfectlight Technology Co., Ltd) as shown in Figure 5c,d (The equipment diagram in Figure S16 in the Supporting Information), showing a linear relationship, which matched well with the computed cumulative charge volume ratio. Moreover, the slops of the curves are 1.98 and 1.93, which is close to the theoretical ratio of two, estimated for the water splitting. Furthermore, the Faradaic efficiency of the NiCo‐nitrides/NiCo_2_O_4_/GF and NiCo_2_O_4_/GF as simultaneous cathode and anode reach ≈100%, respectively in the 1 m KOH. Photographs and videos show that the fibrous electrodes consisted of a bunch of NiCo‐nitrides/NiCo_2_O_4_/GF continuously released small bubbles owing to full use of substrate to achieve all‐round water splitting (Figure S17 and video in the Supporting Information). In general, the electrocatalytic overall water splitting activities of the NiCo‐nitrides/NiCo_2_O_4_/GF were comparable to that of other recently reported overall water splitting electrocatalysts (Table S2, Supporting Information).

**Figure 5:**
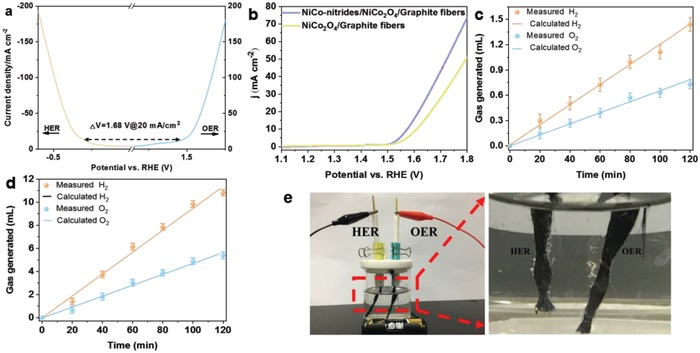
Electrocatalytic activities for overall water splitting in 1 m KOH. a) Steady‐state polarization curves of the NiCo‐nitrides/NiCo_2_O_4_/GF in 1 m KOH for HER and OER; b) Steady‐state polarization for overall water splitting of the NiCo‐nitrides/NiCo_2_O_4_/GF and the NiCo_2_O_4_/GF in two‐electrode configuration; c) O_2_ and H_2_ production volumes as a function of water‐splitting time for the NiCo_2_O_4_/GF// the NiCo_2_O_4_/GF and NiCo‐nitrides/NiCo_2_O_4_/GF// the d) NiCo‐nitrides/NiCo_2_O_4_/GF; e) Photographs showing the electrolyzer with anode and cathode both comprising of NiCo‐nitrides/NiCo_2_O_4_/GF.

We noted that NiCo‐nitrides/NiCo_2_O_4_/GF does not only perform excellent HER and OER performance in alkaline electrolyte but shows a response towards HER and OER in both acid and neutral media. The HER activities of the NiCo_2_O_4_/GF and the NiCo‐nitrides/NiCo_2_O_4_/GF electrode in acid were evaluated in N_2_‐saturated 0.5 m H_2_SO_4_ with a scan rate of 5 mV s^−1^. The NiCo‐nitrides/NiCo_2_O_4_/GF exhibits a lower overpotential of 432 mV than that of NiCo_2_O_4_/GF (573 mV) at a current density of 10 mA cm^−2^ (**Figure** **6**). Although the overpotentials of the NiCo‐nitrides/NiCo_2_O_4_/GF and the NiCo_2_O_4_/GF are higher than that of Pt/C (20 mV), the Tafel plots of the NiCo‐nitrides/ NiCo_2_O_4_/GF (68 mV dec^−1^) and the NiCo_2_O_4_/GF (88 mV dec^−1^) are close to that of Pt/C (32 mV dec^−1^). Moreover, Figure 6c shows that the NiCo‐nitrides/NiCo_2_O_4_/GF has the smaller semicircular Nyquist plots, indicating a smaller charge transfer resistance than that of NiCo_2_O_4_/GF. We also examined the electrocatalytic of the NiCo‐nitrides/NiCo_2_O_4_/GF and the NiCo_2_O_4_/GF for OER in O_2_‐saturated 0.5 m H_2_SO_4_ at a scan rate of 5 mV s^−1^. The NiCo‐nitrides/NiCo_2_O_4_/GF displays a higher electrocatalytic activity with a small overpotential of 460 mV at a current density of 10 mA cm^−2^ than that of NiCo_2_O_4_/GF (490 mV), which is comparable to commercial RuO_2_ (390 mV). The Tafel plots of the NiCo‐nitrides/NiCo_2_O_4_/GF (73 mV dec^−1^) and the NiCo_2_O_4_/GF (82 mV dec^−1^) are close to that of RuO_2_ (67 mV dec^−1^) (Figure 6e). Notably, the OER activities of transition metal‐based compounds in acid media have received little attention.^23^ Transition metal‐based compounds such as Co‐based, and Ni‐based are not stable in acidic media due to the quick corrosion according to the previous literature.^24^ Here, NiCo‐nitrides/NiCo_2_O_4_/GF shows a response toward HER and OER activities in acidic medium, which probes a new approach to attempt the transition metal‐based compounds performing HER and OER simultaneously in acid medium and some mechanism inside the process.

**Figure 6:**
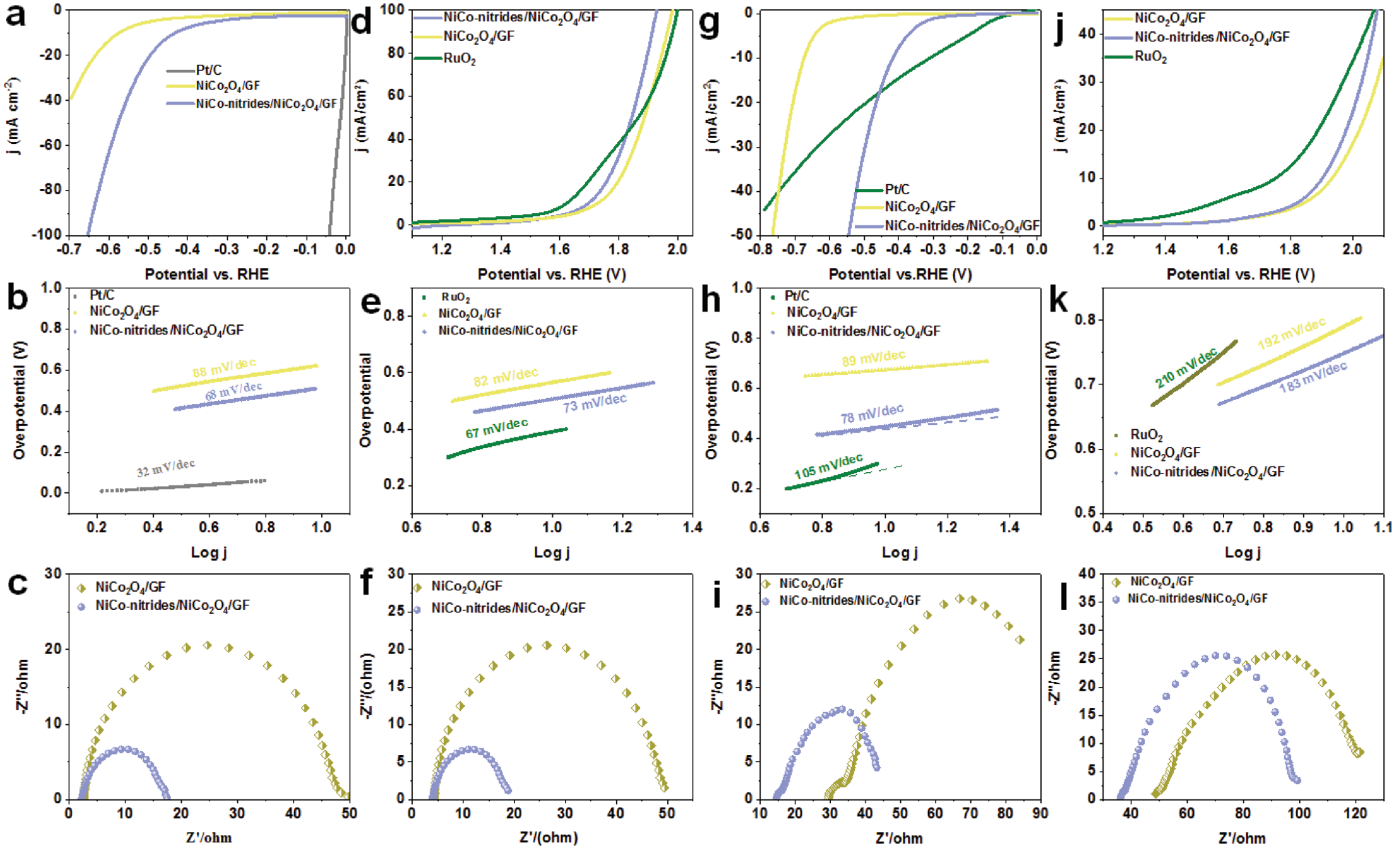
Electrocatalytic HER and OER overall water splitting activities in 0.5 m H_2_SO_4_ and 1 m PBS (pH = 7). a–f) HER and OER activities of the catalysts in 0.5 m H_2_SO_4_; g–l) HER and OER activities of the catalysts in 1 m PBS(pH = 7). a) Linear sweep voltammetry (LSV) curves for HER without IR‐corrected; b) Tafel plots of all samples in 0.5 m H_2_SO_4_; c) EIS Nyquist plots at −0.45 V; d) LSV curves for OER without IR‐corrected; e) corresponding Tafel plots; f) EIS at 1.65 V for different electrocatalysts; g) Linear sweep voltammetry (LSV) curves for HER without IR‐corrected; h) Tafel plots of all samples in 1 m PBS(pH = 7); i) EIS Nyquist plots at −0.65 V; g) LSV curves for OER without IR‐corrected; k) corresponding Tafel plots; l) EIS at 1.85 V for different electrocatalysts.

The transition metal oxides are typically highly prone to corrosion in strongly acidic conditions. The unique hybrid structures of NiCo‐nitrides/NiCo_2_O_4_ with three specifically metallic components can overcome this issue to maintain excellent performance and stability. Furthermore, the coordination between Ni and Co in the heterostructures contributes to resist corrosion according to the recent review related to transition metal‐based water oxidation.^25^ The introduction for nitrides of N atom strengthen this coordination further boosting the electrochemical stability. Most electrocatalysts operating in acid media only exhibit monofunctional activities toward either HER or OER (Table S3, Supporting Information). We not only achieved satisfactory bifunctional electrocatalytic performance from the same materials but also developed a new method by introducing the hybrid structures to boost the electrocatalytic activities of the synthesized electrocatalysts. These findings should attract broad attention from researchers focused on bifunctional electrocatalysts for overall water splitting.

The development of OER and HER electrocatalysts operating in neutral medium is of critical importance owing to safety consideration and to reduce the costs for the water splitting. Despite of many efforts, the current density is still worth enhancing and the overpotentials need to be lowered. For the purpose, we investigated the HER and OER activities of the NiCo‐nitrides/NiCo_2_O_4_/GF in 1 m PBS (pH = 7). The HER activities of the NiCo_2_O_4_/GF and the NiCo‐nitrides/NiCo_2_O_4_/GF electrodes were evaluated in N_2_‐saturated 1 m PBS (pH = 7) at a scan rate of 5 mV s^−1^. The comparison in Figure 6g displays that the NiCo‐nitrides/NiCo_2_O_4_/GF exhibits inferior HER activity with overpotential of 418 mV to afford current density of 10 mA cm^−2^. Conversely, the NiCo_2_O_4_/GF requires an overpotential of 674 mV, which indicates the NiCo‐nitrides/NiCo_2_O_4_ compounds features improved catalytic activities. The corresponding Tafel plots of 78 and 89 mV dec^−1^ for the NiCo‐nitrides/NiCo_2_O_4_/GF and the NiCo_2_O_4_/GF are shown in Figure 6h, respectively. Although the overpotentials of 314 mV for Pt/C is better than that of the synthesized catalysts, the Tafel slop of 105 mV dec^−1^ for Pt/C is inferior to that of the NiCo‐nitrides/NiCo_2_O_4_/GF and the NiCo_2_O_4_/GF. The EIS curves confirm that the improved electron transfer for the NiCo‐nitrides/NiCo_2_O_4_/GF with smaller semicircular arc compared with that of NiCo_2_O_4_/GF. We further investigated the OER performance of the NiCo_2_O_4_/GF and the NiCo‐nitrides/NiCo_2_O_4_/GF electrodes compared with that of commercial RuO_2_ in O_2_‐saturated 1 m PBS (pH = 7) at a scan rate of 5 mV s^−1^. To attain a current density of 10 mA cm^−2^, the NiCo‐nitrides/NiCo_2_O_4_/GF and the NiCo_2_O_4_/GF require an overpotential of 673 and 703 mV, which rivals that of the commercial RuO_2_ (520 mV) (Figure 6j). The Tafel plots of the NiCo‐nitrides/NiCo_2_O_4_/GF, the NiCo_2_O_4_/GF and commercial RuO_2_ are 183, 192, and 210 mV dec^−1^, respectively (Figure 6k). The EIS results shown in Figure 6l confirm the improved electron transfer in the NiCo‐nitrides/NiCo_2_O_4_/GF through evaluation of the size of semicircular plots. The above results suggest that electrocatalytic performance of the NiCo‐nitrides/NiCo_2_O_4_/GF is superior to that of some recently reported electrocatalysts in neutral media for HER and OER (Table S3, Supporting Information).

The aforementioned data indicates that NiCo‐nitrides/NiCo_2_O_4_/GF has remarkable OER and HER activities and superior stability over a wide pH range. We attribute the remarkable OER and HER activities of NiCo‐nitrides/NiCo_2_O_4_/GF to the interfaces formed by the nitridation of NiCo_2_O_4_. To obtain further insight, we performed the first‐principles density function theory (DFT) calculations to determine the Fermi level and the spin resolved total electron density of states (TDOS) for Ni_3_N, NiCo_2_O_4_, and CoN, respectively using Vienna ab initio simulation package known as the VASP code. All three compounds are metallic in character and the filled area shows the occupied state and the energy of the highest occupied state is the Fermi level. The metallic character ensures the efficient electron transfer. Their Fermi level are 0.44, 0.16, and 2.4 eV respectively (**Figure** **7**a). At the NiCo_2_O_4_/CoN interface, electron transfer from NiCo_2_O_4_ to CoN occurs to align the Fermi levels of the two components, resulting in a contact electric potential (CEP) of ≈2.24 V across the interface. Similar processes also occur at the NiCo_2_O_4_/Ni_3_N interface with a CEP of ≈0.28 V. This CEP acts as an additional electric potential versus the reversible hydrogen electrode, which offers interface electric field that facilitates electrochemical reactions (Figure 7b). Notably, we investigated the functionality of Ni_3_N and CoN derived from nitridation of NiCo_2_O_4_ by comparing the relationships between electron transfer and the Fermi level for the oxides with oxides/nitrides. The above analysis is consistent well with experimental observations on the intrinsic electrocatalytic activity and the electrochemical reaction mechanism (see Figure S18 in the Supporting Information for more details).

**Figure 7:**
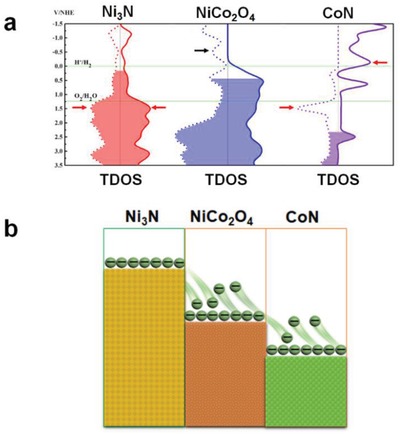
a) Spin resolved total electron density of states (TDOS) for Ni_3_N, NiCo_2_O_4_, and CoN. The filled area is the occupied state and the energy of the highest occupied state is the Femi level. b) Schematics of interface electric field effect contributing to electrons transfer.

The NiCo‐nitrides/NiCo_2_O_4_/GF exhibited distinct activities toward HER and OER over a wider pH range. These bifunctional electrodes for HER and OER were fabricated by in situ electrodeposition and in situ modification. Metallic NiCo_2_O_4_ with bifunctional activity for HER and OER was selected as the basic precursor. After nitrogenation, CoN and Ni_3_N were formed in situ and enhanced the OER or HER performance. The metallic properties of these materials, and the valence and electronic states of the double‐metal nitrides, promoted electrostatic adsorption of H^+^ or OH^−^ ions and H_2_O molecule, and reduced the energy barrier for transitions of Ni and Co from lower to higher valence states. These findings contrast with those of single‐metal nitrides and pure metal oxides as previously reported by Zhang et al.[[qv: 12b]] Of particular interest, the p orbitals of N are continuous, producing a covalent interaction between Ni, Co, and N in the metal nitrides. This type of metallic behavior is different from that in metals and can help to resist erosion from acid media. The interface electric field effect at the highly‐coupled interfaces of the oxides and nitrides also drives electron transfer, inducing electron flow until work function equilibrium is reached, further lowering the overpotentials of the electrocatalysts. The many edges of the nanoflakes, the walls, and the pore structures give the nanostructures a large surface area and many electroactive sites for redox reactions. A large number of nanospheres assembled on the surface of the interconnected nanoflakes, forming a unique 3D beaded fabric‐like nanostructure, which offered a pathway for rapid electron‐transport from the interface of the electrodes and electrolyte to the excellent intrinsic electrically conductive graphite fibers. The unique 3D structures, complex combination of components, and metallic behavior, give our NiCo‐nitrides/NiCo_2_O_4_/GF catalysts bifunctional electrocatalytic activities toward HER and OER with remarkable performance, superior stability, and good reaction kinetics. The above advantages for electrocatalytic activities make our NiCo‐nitrides/NiCo_2_O_4_/GF effective all‐round electrodes, which are particularly effective for overall water splitting over a wide pH range.

We have developed a 3D electrode configuration composed of metallic NiCo‐nitrides/NiCo_2_O_4_/GF as pH‐universal bifunctional electrocatalysts toward HER and OER for overall water splitting. The NiCo‐nitrides/NiCo_2_O_4_/GF displayed an overpotential of 71 and 183 mV at 10 mA cm^−2^ for HER and OER in 1 m KOH, respectively, as well as superior stability. Furthermore, for electrocatalytic overall water splitting of the NiCo‐nitrides/NiCo_2_O_4_/GF in 1 m KOH, the overall potential window was 1.68 V at 20 mA cm^2^ and the Faradaic efficiency reached to ≈100%. The NiCo‐nitrides/NiCo_2_O_4_/GF exhibits a response in acid and neutral media. First‐principles simulations reveal that the metallic behaviors of NiCo_2_O_4_ and NiCo‐nitrides/NiCo_2_O_4_, and the interface electric field effect at the interface of NiCo‐nitrides/NiCo_2_O_4_ are the key factors for improving the electron transfer efficiency. This work opens new possibilities for the use of double‐metal nitrides/oxides heterostructures for overall water splitting.

## Conflict of Interest

The authors declare no conflict of interest.

## Supporting information

Supplementary

Supplementary
