# Supplementary material for: Promotion of Overall Water Splitting Activity Over a Wide pH Range by Interfacial Electrical Effects of Metallic NiCo‐nitrides Nanoparticle/NiCo2O4 Nanoflake/graphite Fibers
Source: Adv Sci (Weinh). 2019 Jan 15;6(5):1801829. doi: 10.1002/advs.201801829 (PMC6402402; doi:10.1002/advs.201801829)
Supplement: Supplementary file 1 — Supplementary [file ADVS-6-1801829-s002.pdf]

Copyright WILEY-VCH Verlag GmbH & Co. KGaA, 69469 Weinheim, Germany, 2019.

Supporting Information

for *Adv. Sci.,* DOI: 10.1002/advs.201801829

Promotion of Overall Water Splitting Activity Over a Wide pH Range by Interfacial Electrical Effects of Metallic NiCo-nitrides Nanoparticle/$NiCo_2O_4$ Nanoflake/graphite Fibers

Zhihe Liu, Hua Tan, Daobin Liu, Xiaobiao Liu, Jianping Xin, Junfeng Xie, Mingwen Zhao, Li Song, Liming Dai, and Hong Liu**

Copyright WILEY-VCH Verlag GmbH & Co. KGaA, 69469 Weinheim, Germany, 2016.

Supplementary Information

Promotion of overall water splitting activity over a wide pH range by interfacial electrical effects of metallic NiCo-nitrides nanoparticle/$NiCo_2O_4$ nanoflake/graphite fibers

Zhihe Liu, Hua Tan, Daobin Liu, Xiaobiao Liu, Jianping Xin, Junfeng Xie , Mingwen Zhao, Li Song, Liming Dai* and Hong Liu*

Z. H. Liu& Prof. H. Liu
Institute for Advanced Interdisciplinary Research (IAIR), University of Jinan, Shandong 250022 (China).
State Key Laboratory of Crystal Materials, Shandong University, Jinan, Shandong 250100 (China). Email: hongliu@sdu.edu.cn.

Dr. T. H& J. P. Xin
State Key Laboratory of Crystal Materials, Shandong University, Jinan, Shandong 250100 (China).

Prof. L. M. Dai
Center of Advanced Science and Engineering for Carbon (Case4carbon), Department of Macromolecular Science and Engineering, Case Western Reserve University, 10900 Euclid Avenue, Cleveland, OH 44106, USA. Email: liming.dai@case.edu.

Prof. J. F. Xie
College of Chemistry, Chemical Engineering and Materials Science, Shandong Normal University, Jinan 250014 (China)

D. B. Liu& Prof. L. Song
National Synchrotron Radiation Laboratory CAS Hefei Science Center University of Science and Technology of China Hefei, Anhui 230026, P. R. China

Dr. X. B. Liu& Prof. M. W. Zhao
School of Physics and Microelectronics, Shandong University Jinan, Shandong 250100 (China)

I Experimental

II Supplementary Results

III Supplementary Videos

I Experimental

1.Experimental Section

Synthesis of $NiCo_2O_4$ supported on graphite fibers: Fabrication of the 3D $NiCo_2O_4$ Precursor Electrodes: The 3D $NiCo_2O_4$ precursor electrodes were fabricated by a simple electrodeposition-thermal decomposition in atmosphere method. In a typical procedure, a hank of graphite fibers (5 μm in diameter, 2 cm in length) were cleaned with acetone, ethanol and deionized water for 10 min in sequence. After that, the electrodeposition was performed in a standard three-electrode quartz cell consisting of the graphite fibers working electrode, a platinum plate counter electrode and a saturated calomel reference electrode (SCE) at room temperature. The bimetallic (Ni, Co) hydroxide precursor was electrodeposition upon graphite fibers in an 8 mM $Co(NO_3)_2·6H_2O$ and 4 mM $Ni(NO_3)_2·6H_2O$ aqueous mixed electrolyte using an Electrochemical Workstation (Chen Hua CHI660 E). The electrodeposition potential is -1.0V (vs. SCE). After electrodeposition for eight minutes, the graphite fibers were washed several times by deionized water and absolute ethanol, and finally dried in air. Then the sample was put in a quartz tube and calcined at 400 ℃ for 2 h to be transferred into ultrathin mesoporous $NiCo_2O_4$ nanoflakes. In average, 0.26 mg of $NiCo_2O_4$ nanoflakes were grown per graphite fibers (2cm in length), carefully weighed after calcination.

Synthesis of NiCo-nitrides/ $NiCo_2O_4$ supported on graphite fibers: The 3D $NiCo_2O_4$ precursor electrodes were calcined at 400 ℃ in NH_3 atmosphere for 2 h to be partially transferred into NiCo-nitrides.

2.Preparation of Pt/C (20wt%) and RuO_2 electrodes: A 1 mL homogeneous catalyst ink was obtained by sonication for 20 min, consisting of 5mg Pt/C or RuO_2, 20 μL Nafion solution (5%) and 980 μL and absolute ethanol. All the catalyst ink was dropped on the glassy carbon electrode (5 mm diameter with loading of ~0.5 mg cm^{-2})

3.Characterization: X-ray power diffraction (XRD) patterns were recorded on a Bruke D8 Advance Power X-ray diffractometer at 40 kV and 40 mA for monochromatized Cu Kα (λ=0.15406 nm). Field emission scanning electron microscopy (FESEM, HITACHI S-4800) and NoVaTM Nano SEM 250 were used to observe the morphology and size of the synthesized samples. The transmission electron microscopic (TEM) images were acquired with a JEOL JEM 2100 microscope operating at 200 kV. The chemical components were investigated by the energy dispersive X-ray spectroscopy (EDX). X-ray photoelectron spectroscopy (XPS) was performed using an ESCALAB 250. The absorption spectra of Ni and Co K-edge were collected in transmission mode using a Si (111) double-crystal monochromator at the X-ray absorption fine structure (XAFS) station of the 1W1B beamline of the Beijing Synchrotron Radiation Facility (BSRF).

4. Electrochemical tests

The electrochemical measurements were performed on a CHI660E electrochemical workstation with a three-electrode cell. The working electrode was NiCo-nitrides/$NiCo_2O_4$/GF (loading: ~0.3 mg) and $NiCo_2O_4$/GF (loading: ~0.26 mg), the counter electrode was platinum plate, and the reference electrode was a saturated Ag/AgCl (3.3 M KCl) electrode. The linear sweep voltammetry curves were recorded at a scan rate of 5 mV s^{-1} in deaerated KOH (1M), H_2SO_4(0.5M) and PBS (1M pH=7) as the electrolyte without IR-corrected. The electrochemical impedance spectroscopy (EIS) measurements were conducted over a frequency range 0.01-10^5 Hz. The long-term durability tests were carried out using the chronopotentiometric measurements. For the geometric area, we tied the hank of graphite fibers tightly with lines and we measured the side area of the graphite fibers as bundle cylinder substrate as geometric area to further estimate the current density.

4.1 Potential transformation between Ag/AgCl and RHE

All potentials reported are calibrated to reversible hydrogen electrode RHE by

$$E_{(RHE)}=E_{(Ag/AgCl)}+0.197\text{ V}+0.059*pH$$

The overpotentials (η) were obtained from:

$$=E_{(RHE)}-1.23\text{ V}$$

And the current density (j) were normalized by geometric surface area. All the

presented curves are the steady-state ones after several cycles.

4.2 Electrochemical active surface area

The capacitive currents are measured in a potential range where no faradic processes are observed. We sweep the potential between 0.1~0.2 V vs RHE at different scan rates. The difference in current density variation($\Delta j = j_a - j_c$) at the potential of 0.15 V vs RHE plotted against scan rate are fitted to estimate the electrochemical double-layer capacitances (C_{dl}), which can be used to estimate the electrochemically active surface area (EASA).

4.3 Faradic efficiency

The Faradic efficiency reflects the utilization efficiency of electron in HER and OER process. For OER (or HER) process, the Faradic efficiency can be obtained by calculating the radio of the experimentally produced O_2 (H_2) amount(n_{O2}) to the theoretical produced O_2 amount($n_{O2'}$). Specifically, under a constant oxidation current (I) within a certain time (t), the experimentally produced O_2 amount can be measured by Automatic online trace gas analysis system (Labsolar 6A supplied by Beijing Perfectlight Technology Co., Ltd.)-gas chromatography (Perfect Light GC7806). Thus, the Faradic efficiency can be calculated as following:

$$\text{Faradic efficiency} = n_{O_2} / n_{O_2'} = 4F\, n_{O_2} / It$$

(or Faradic efficiency= n_{H_2}/ $n_{H_2'}$=4F n_{H_2}/It

5.Computational formulas:

Our first-principles calculations were performed within density-functional theory (DFT) using the Vienna ab initio simulation package known as the VASP code.[1] The projector augmented wave method (PAW) [2]was used to describe the electronic-ion interaction. The energy cutoff of the plane waves was set to 450 eV with an energy precision of 10^{-5} eV. The electron exchange–correlation function was treated using a generalized gradient approximation (GGA) in the form proposed by Perdew, Burke, and Ernzerhof (PBE).[3] The method of local density approximation (LDA)+U, The U value for Co and Ni taken from the previous work are 6.7 and 7.1 respectively.[4], The Monkhorst-Pack[5] k-point meshes for the Brillouin zone (BZ) sampling are well converged for each system . Both atomic positions and lattice vectors were fully optimized using the conjugate gradient (CG) algorithm until the maximum atomic forces were less than 0.01 eV/Å. The equilibrium lattice constant for each system is donated in Figure S18. To compare the fermi level of CoN, Ni_3N and $NiCo_2O_4$, the fermi level relative to electrostatic energy in vacuum is thus calculated. The energy of core states referred to electrostatic energy in vacuum for center atom of slab which is thick enough to describe that of bulk is obtained, and consequently the fermi level compared with electrostatic energy in vacuum is gotten by align the energy of core states of slab and bulk. The fermi level for $NiCo_2O_4$, Ni_3N and CoN is 0.44, 0.16 and 2.4 eV vs NHE respectively.

II Supplementary Results

S1 SEM image and XRD pattern of bulk CoN

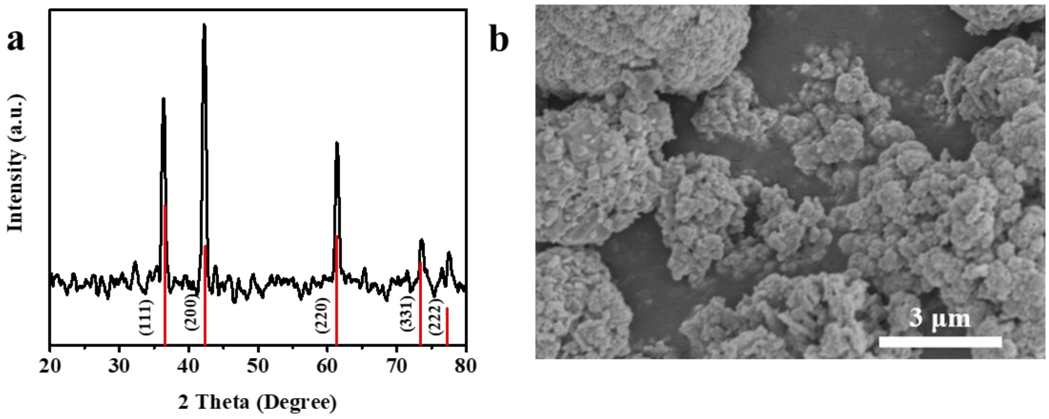

Figure S1a shows that the XRD pattern of prepared CoN was well indexed to the characteristic peaks of CoN (JPCDS No.83-0831). Furthermore, the morphology of CoN consisted of nanoparticles investigated by SEM in Figure S1b. The prepared CoN was used as standard reference sample in XAFS measurements

S2 SEM image and XRD pattern of bulk Ni_3N

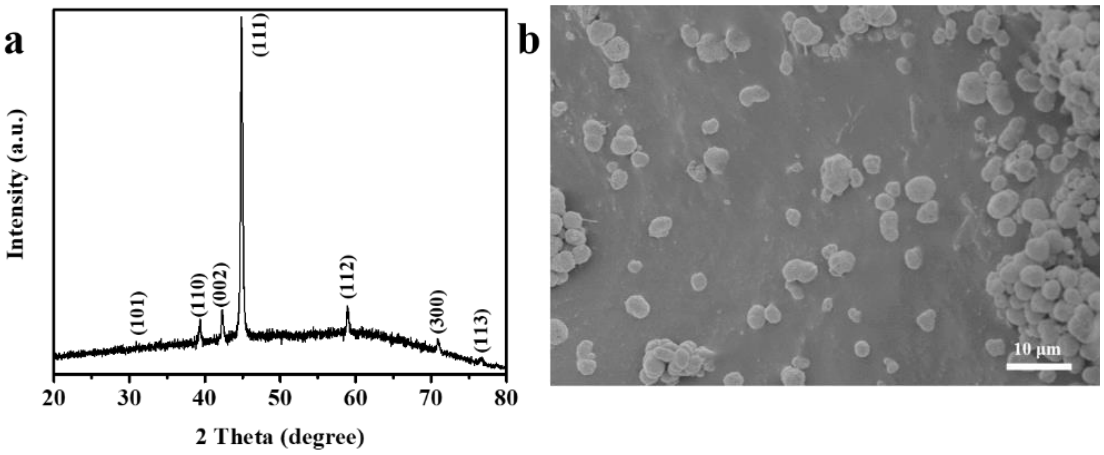

Figure S2a shows that the XRD pattern of prepared Ni_3N was well indexed to the characteristic peaks of Ni_3N (JPCDS No.89-5144). Furthermore, the morphology of

Ni_3N consisted of nanoparticles investigated by SEM in Figure S2b. The prepared Ni_3N was used as standard reference sample in XAFS measurements.

S3 Raman spectrum of graphite fibers

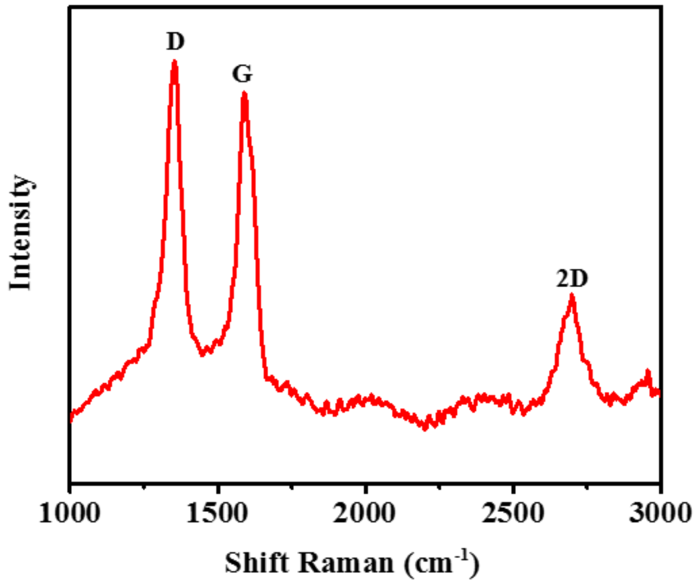

Figure S3 The Raman spectrum of bare graphite fibers. As is shown in Figure S3, the Raman spectrum of bare graphite fibers composited of three characteristic peaks at 1350 cm^{-1},1592 cm^{-1} and 2692 cm^{-1}, respectively, which is corresponding to D-band, G-band and 2D-band, respectively. To our knowledge, G-band is related to in-plane vibration of carbon atoms in sp2 flat, 2D-band stand for the number layers and stacking order of graphene. The conductivity of carbon-based materials depends on the ratio of I_D/I_G demonstrates the degree of graphitization. The ratio of I_D/I_G in the Figure S3 was 1.06, indicating the high degree of graphitization, which further influences the electron transfer from the sample to the substrates.

S4 SEM images of NiCo-hydroxides assembled on graphite fibers.

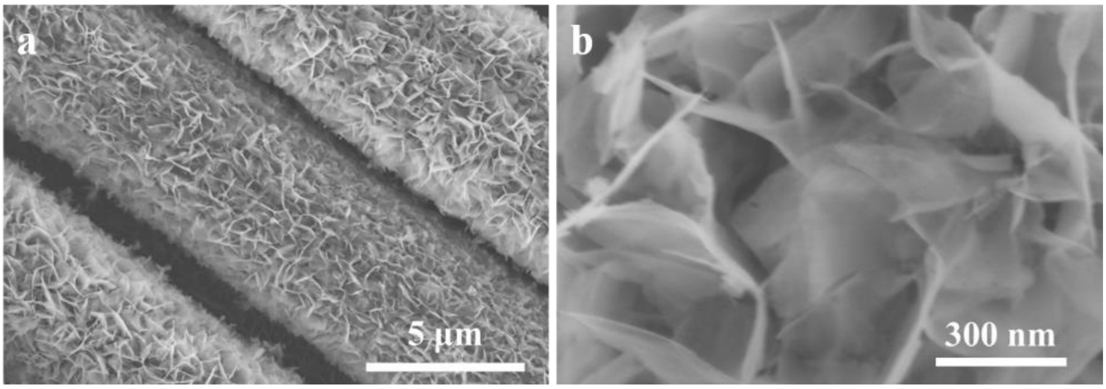

Figure S4 SEM images of NiCo-hydroxides assembled on graphite fibers show that a layer of mixed nickel-cobalt hydroxides was assembled on the surface of a bundle of microscale graphite fibers by a facile co-electrodeposition method in a mixed solution of cobalt and nickel nitrate. To further verify the structures of the nanoflakes, the high-resolution SEM image for Figure S4b shows that the urchin nanoflakes with the thickness of ~10 nm were interconnected with each other. Combined with the SEM images of $NiCo_2O_4$ assembled on graphite fibers, it can be confirmed that nanopores occurred during the pyrolysis for NiCo-hydroxides.

S5 SEM image of the $NiCo_2O_4$ on graphite fibers after nitrogenation for 4h

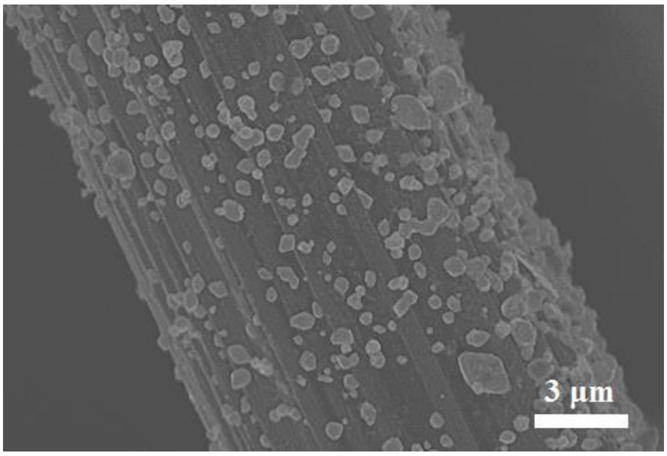

Figure S5 shows the SEM image of $NiCo_2O_4$ on graphite fibers after nitrogenation for 4h. Clearly, the nanoflakes mostly melt after 4h in NH_3 atmosphere and fell off the graphite fibers. Thus, we choose nitrogenation for 2h reasonably.

S6 The cross-sectional views after electrodeposition.

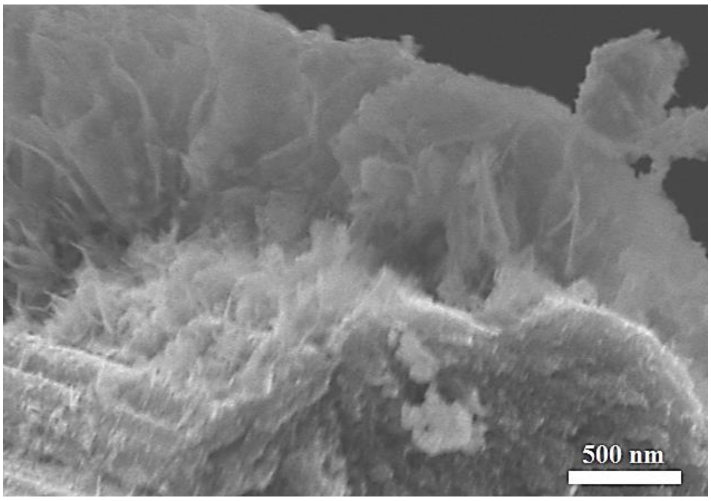

From the Figure S6, we can see clearly that the nanoflakes were tightly attached on the graphite fibers forming 3D nanostructures. Furthermore, the thickness of ~500 nm was demonstrated here.

S7 TEM image of $NiCo_2O_4$ derived from the $NiCo_2O_4$/GF sample.

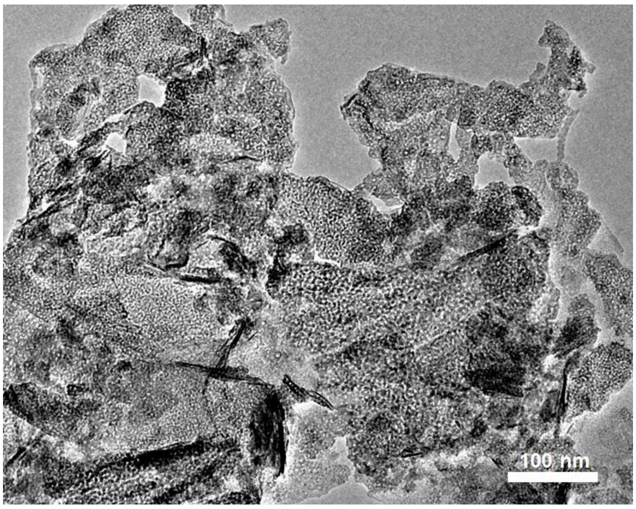

The TEM image of $NiCo_2O_4$ confirms the porous structures. Large amount of edges of the nanoflakes and the wall of pore endow the nanostructures abundant open space and electroactive sites for redox reaction.

S8 HRTEM image of $NiCo_2O_4$ derived from the $NiCo_2O_4$/GF sample.

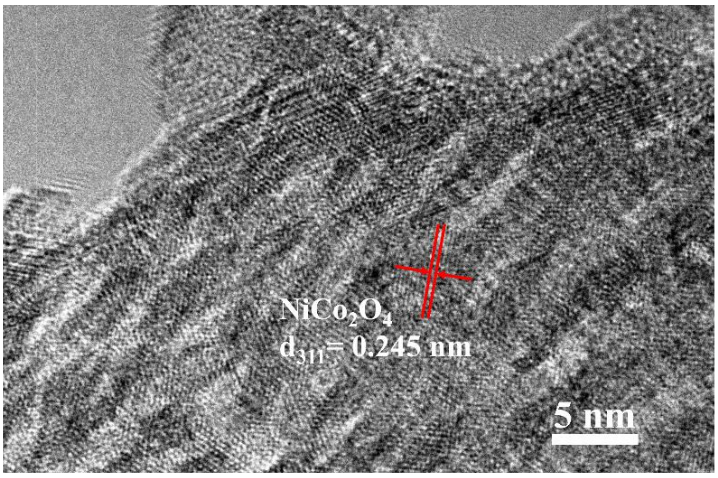

High-resolution TEM (HRTEM) image of $NiCo_2O_4$ derived from the synthesized electrode sample reveals that the clear lattice fringes of one part are 0.245 nm, which is corresponding to the (311) plane of $NiCo_2O_4$

S9 XPS peak of N1s spectra of NiCo-nitrides/ $NiCo_2O_4$/GF on graphite fibers

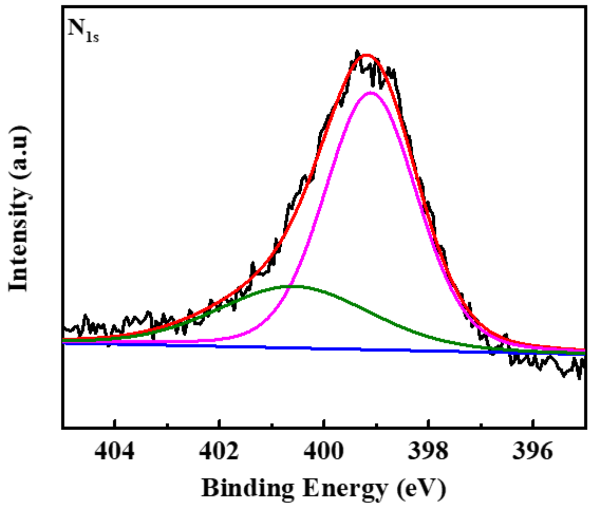

For N1s spectrum in Figure S9, the characteristic peak was located at around 399 eV, which can be assigned to the nitrogen in a metal nitride.

S10 Chronopotentiometry of the NiCo-nitrides/$NiCo_2O_4$/GF and $NiCo_2O_4$/GF for HER

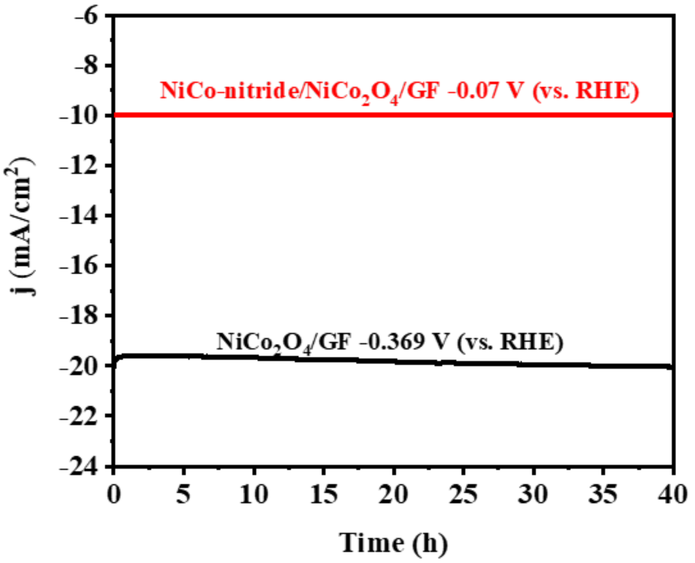

Figure S10 Chronopotentiometry of the NiCo-nitrides/$NiCo_2O_4$/GF and $NiCo_2O_4$/GF for HER. Figure S10 show the results of a stability test conducted for the NiCo-nitrides/$NiCo_2O_4$/GF and the $NiCo_2O_4$/GF at a constant potential of -0.07 V (vs

RHE) and -0.369 V (vs RHE), respectively. Almost no decrease in current density occurred over 40 h for the NiCo-nitrides/$NiCo_2O_4$/GF by continuous chronoamperometric response (i-t) under alkaline condition.

S11 SEM image of NiCo-nitrides/$NiCo_2O_4$/GF after 40h by continuous chronoamperometric response (i-t) under alkaline condition

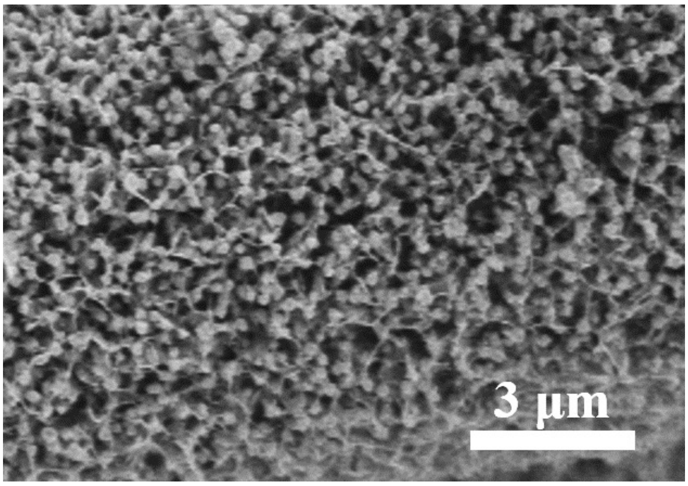

The morphology of the NiCo-nitrides/$NiCo_2O_4$/GF hardly changed, which indicates the good stability of the NiCo-nitrides/$NiCo_2O_4$/GF.

S12 Equivalent circuit of EIS

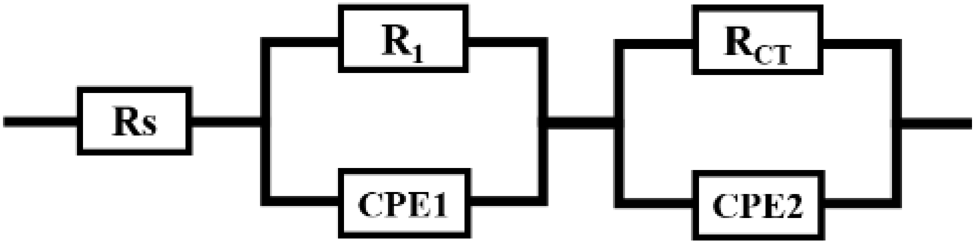

The equivalent circuit consisted of a resistor (Rs) in series with two parallel combinations of a resistor (R1, Rct) and a constant phase element (CPE1, CPE2). This

was used to fit the EIS data that can be evidenced by the semicircles in the high- and low-frequency range. R_s and R_{ct} were determined by electrocatalytic kinetics, may result from the Ohmic resistance arising from the electrolyte as well as the all contact, and charge transfer resistance at the interface between the catalysts and the electrolyte, respectively. It is known to us that small values of R_s correspond to close contact between current collector and catalysts, and that small values of R_{ct} endow the electrocatalysts with rapid charge transfer kinetics.

S13 Chronopotentiometry of the NiCo-nitrides/$NiCo_2O_4$/GF and $NiCo_2O_4$/GF for OER

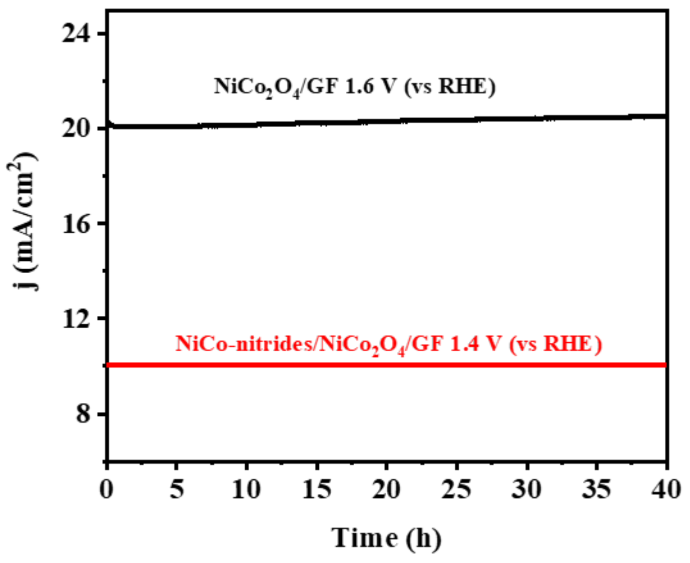

The stability test of the NiCo-nitrides/$NiCo_2O_4$/GF and the $NiCo_2O_4$/GF in Figure S13 was also tested by continuous chronoamperometric response (i-t) under alkaline condition at the applied potential of 1.41 V (vs RHE) and 1.60V (vs RHE), respectively. They show almost negligible degradation during 40 h of continuous operation, which confirms the excellent durability.

Catalyst	Electrolysis test	η (mV)	Tafel (mV/dec)	Electrolyte	References

Table S1 Comparison of the electrocatalytic performance in 1M KOH of bifunctional water splitting non-noble electrocatalysts reported. The current density (j) here is 10 mA cm^{-2}

Ni_3FeN	HER	158	42	1M KOH	Adv. Energy Mater.2016,6,1502585
	OER	280	46		
CoOx@CN	HER	232	115	1M KOH	J. Am. Chem.Soc. 2015, 137
	OER	260	/		
Co-P	HER	94	42	1M KOH	Angew. Chem. Int. Ed. 2015, 54,6251
	OER	345	47		
Ni_5P_4/Ni foil	HER	150	53	1M KOH	Angew. Chem. Int. Ed. 2015, 54,
	OER	140	40	0.5M H_2SO_4	
Ni/NiP	HER	120	58.5	1M KOH	Adv. Funct. Mater.2016,26,3314-3323
	OER	270	73		
$NiCo_2O_4$	HER	110	49.7	1M KOH	Angew. Chem. Int. Ed. 2016, 55, 6290 –6294
	OER	290	53		
MoO_2	HER	27	41	1M KOH	Adv. Mater.2016，28，3785-3790
	OER	260	54		
MoOx/Ni_3S_2/NF	HER	106	90	1M KOH	Adv. Funct. Mater.2016,26,4839-4847
	OER	136	/		
Ni_3S_2/NF	HER	223	/	1M KOH	J. Am. Chem.Soc. 2015, 137,14023
	OER	260	/		
$NiCo_2O_4$/GF	HER	307	58	1M KOH	This work
	OER	308	61		
the NiCo-nitrides/$NiCo_2O_4$/GF	HER	71	35	1M KOH	
	OER	183	56		

Table S2 Comparison of the electrocatalytic performance in 1M KOH of overall water splitting non- noble electrocatalysts reported. The current density (j) here is 10 mA cm^{-2}

catalyst	electrolysis test	η_{10} (mV)	References
NiFe LDH/NF	OWS	470	Science.2014, 345, 1593.
NiSe/NF	OWS	400	Angew. Chem. Int.Ed. 2015, 127, 9483.
Ni_2P	OWS	400	Energy Environ. Sci.2015, 8, 2347.
Ni_5P_4	OWS	470	Angew.Chem. Int.Ed.2015, 54,12361.
Co-P film	OWS	410	Angew. Chem. Int.Ed. 2015, 54, 6251
$NiFeO_x$/CFP	OWS	280	Nat. Commun.2015, 6, 7261.
NiC//NiD-PCC	OWS	620	Energy Environ. Sci. 2016, 9(11):

			3411-3416.
$MoO_x/Ni_3S_2/NF$	OWS	480	Adv. Funct. Mater. 2016, 26, 4839–4847
MoO_2/NF	OWS	300	Adv. Mater. 2016, 28, 3785–3790
$NiCo_2O_4/GF$	OWS	**370**	This work
NiCo-nitrides/$NiCo_2O_4/GF$	OWS	**340**	

Table S3 Comparison of the electrocatalytic performance in 0.5M H_2SO_4 of bifunctional water splitting non-noble electrocatalysts reported. The current density (j) here is 10 mA cm^{-2}

catalyst	electrolysis test	η (mV)	Tafel (mV/dec)	electrolyte	References
WS_2/Graphene	HER	200	58	0.5M H_2SO_4	Angew.Chem. Int.Ed, 2013,52,13751-13754
	OER		80		
FeP NPs	HER	130	67	0.5M H_2SO_4	Nanoscale, 2017,9,3555-3560
	OER	/	/		
Cu-MOF	HER	192	84	0.5M H_2SO_4	Adv. Funct. Mater. 2013, 23, 5363–5372
	OER	/	65		
FeP	HER	58	45	0.5M H_2SO_4	ACS Cata.2014,4,4065-4069
	OER	/	/		
$CoSe_2$-CP	HER	137	40	0.5M H_2SO_4	J. Am. Chem. Soc.2014,136,4897-4900
	OER	/	/		
α-FeNi-S	HER	105	40	0.5M H_2SO_4	J. Am. Chem. Soc.2015,137,11900-11903
	OER	/	/		
1T-MoS_2	HER	/	/	0.5M H_2SO_4	Adv. Mater. Interfaces 2016,3,1500669
	OER	420	282		
C/Co_3O_4	HER	/	/	0.5M H_2SO_4	Nano Energy 2016,25:42-45
	OER	370	84		
Co_3O_4/FTO	HER	/	/	0.5M H_2SO_4	Chem. Mater. 2017, 29, 950−957
	OER	490	80		
$NiCo_2O_4/GF$	HER	573	88	0.5M H_2SO_4	This work
	OER	490	82		
NiCo-nitrides/$NiCo_2O_4/GF$	HER	432	68	0.5M H_2SO_4	
	OER	460	73		

Table S4 Comparison of the electrocatalytic performance in 1M PBS(pH=7) of bifunctional water splitting non-noble electrocatalysts reported. The current density (j) here is 10 mA cm^{-2}

catalyst	electrolysis test	η (mV)	Tafel (mV/dec)	electrolyte	Reference
N_3S_2/NF	HER	170	/	PBS(pH=7)	J. Am. Chem. Soc, 2015, 137(44): 14023-14026.
	OER	260			
CoP_2	HER	/	/	PBS(pH=7)	Nano Letters, 2016, 17(1): 578-583.
	OER	100	129.8		
Co_9S_8	HER	175	/	PBS(pH=7)	JMCA, 2016, 4(18): 6860-6867
	OER	/	/		
Co-P based	HER	/	/	PBS(pH=7)	Angewandte Chemie, 2017, 129(4): 1084-1088
	OER	450	187		
Co/PB@rGO	HER	/	/	PBS(pH=7)	JMCA,2014,2(43):18420-18427.
	OER	400	68		
Co-Bi/Ti	HER	/	/	PBS(pH=7)	JMCA,2017
	OER	469	131		
$CoSe_2$/CoO	HER	200	131	PBS(pH=7)	Advanced Science, 2016, 3(6)
	OER	510	183		
CoP_4N_2	HER	890	/	PBS(pH=7)	Energy & Environ. Science, 2014, 7(1): 329-334.
	OER	/	/		
FeP NAs	HER	218	146	PBS(pH=7)	ACS Catal. 2014, 4, 4065−4069
	OER	/	/		
$NiCo_2O_4$/GF	HER	674	89	PBS(pH=7)	This work
	OER	703	192		
NiCo-nitrides/$NiCo_2O_4$/GF	HER	418	78	PBS(pH=7)	
	OER	673	183		

S14 Long-term controlled current density electrolysis of the NiCo-nitrides/$NiCo_2O_4$/GF for overall water splitting in 1M KOH

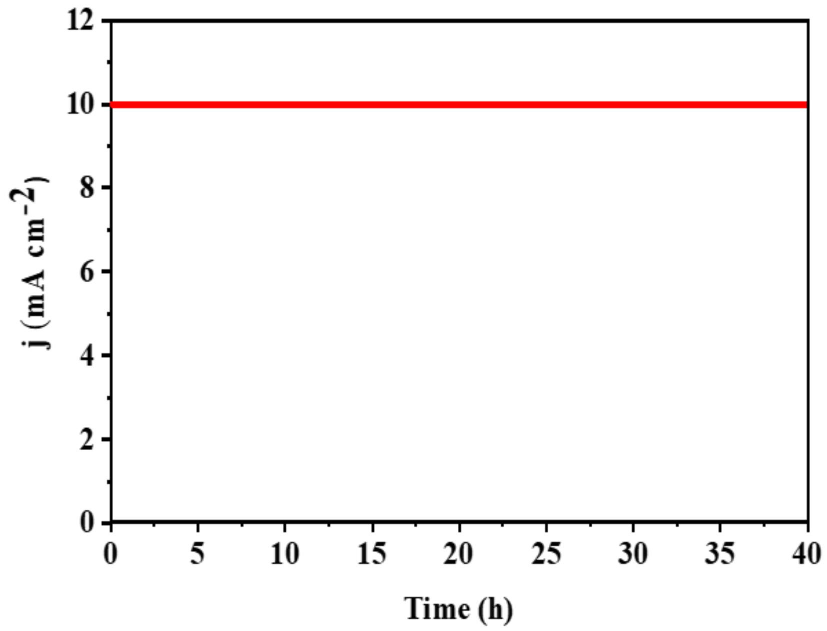

The durability test was maintained at 10mA/cm^2 in 1 M KOH (Figure S14), which also show hardly never degrade after 40 h. It demonstrates the good stability of the NiCo-nitrides/$NiCo_2O_4$/GF for overall water splitting in 1M KOH

S15 Electrochemical active surface area

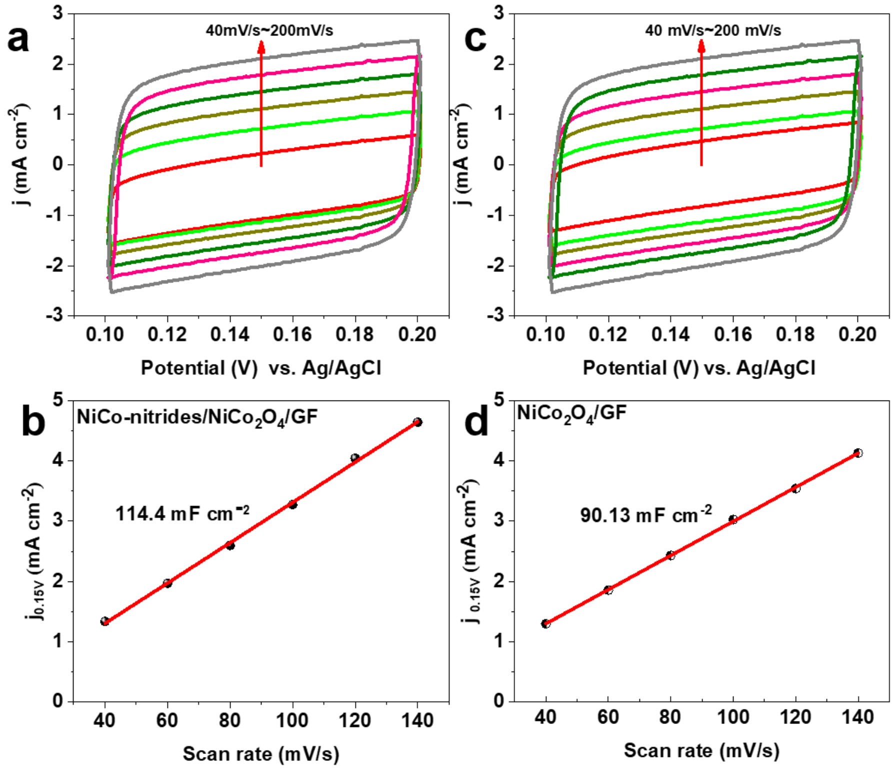

Figure S15 (a) CV curves for the NiCo-nitrides/$NiCo_2O_4$/GF; (b) Charging current density with different scan rates corresponding to the NiCo-nitrides/$NiCo_2O_4$/GF; (c) CV curves for $NiCo_2O_4$/GF; (d) Charging current density with different scan rates corresponding to $NiCo_2O_4$/GF

To further demonstrate the enhanced performance of synthesized samples, we compared the electrochemical surface area (ECSA) of the NiCo-nitrides/$NiCo_2O_4$/GF and the $NiCo_2O_4$/GF by measuring the electrochemical double-layer capacitance (C_{dl}). As we can see in Figure S15, the NiCo-nitrides/$NiCo_2O_4$/GF delivered a higher C_{dl} (114.4 mF cm^{-2}) than $NiCo_2O_4$/GF (90.13 mF cm^{-2}), which is proportional ECSA. As a function of previous reports[6], the activity enhancement arose from the increased

electrochemical surface area, which indicating the NiCo-nitrides/$NiCo_2O_4$/GF possess higher exposure pf active sites

S16 The diagram of the Automatic online trace gas analysis system-gas chromatography (Labsolar-6A, Beijing Perfectlight Technology Co., Ltd)

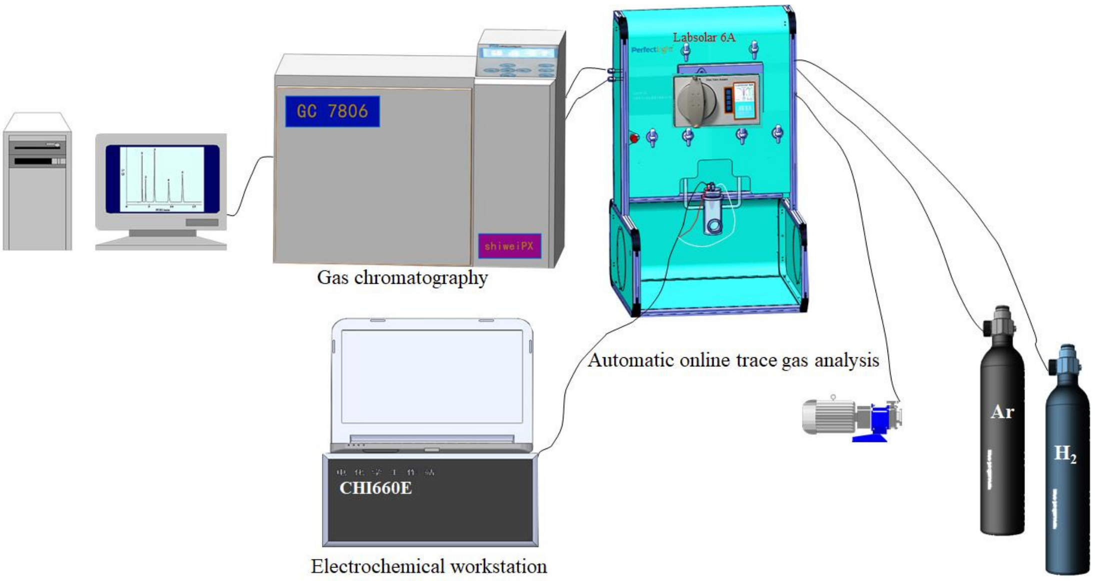

S17 Photographs showing an electrolyzer, whose anode and cathode both comprised NiCo-nitrides/$NiCo_2O_4$/GF for all-round water splitting

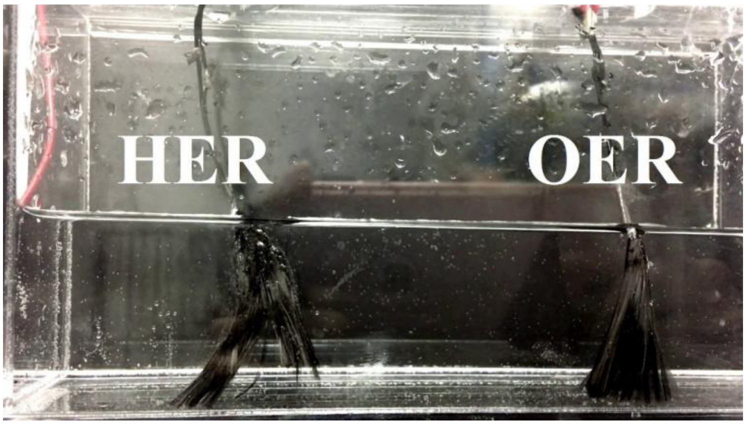

From Figure S17, we can clearly see that the bubbles of H_2 and O_2 released at the

anode and cathode. The diffuse graphite fibers as the substrate guarantee the all-round electrocatalytic water splitting.

S18 Crystal structures for Ni_3N, CoN and $NiCo_2O_4$

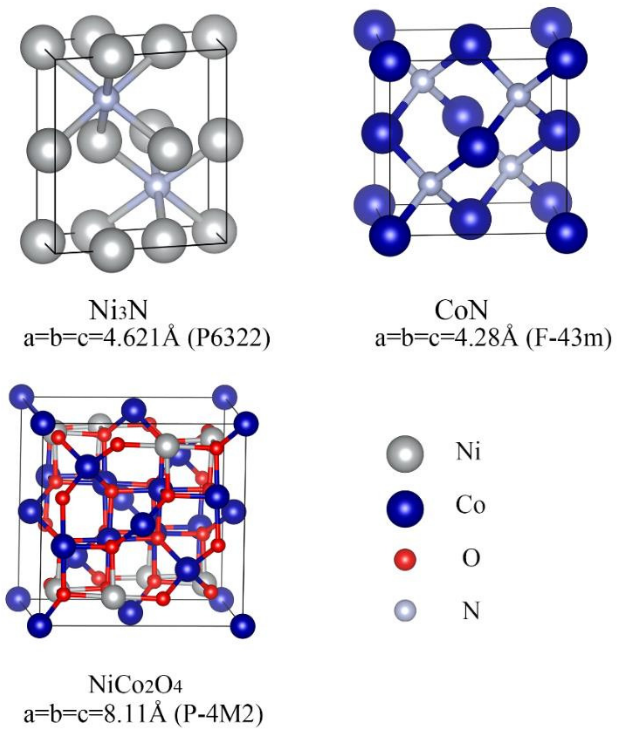

Figure S18 The crystal structures for Ni_3N, CoN and $NiCo_2O_4$, respectively. The gray ball represents for Ni atoms, blue ball for Co atoms and red ball for oxygen atoms.

It is well accepted that the Femi level would change in the effect of electric potential. [1-6] The Femi level tends to migrate towards the high-energy level direction when applied negative potential. When the only existence of $NiCo_2O_4$ occurred in HER process, electrons flow from the elevatory Femi level that contains lots of TDOS marked by black arrows of $NiCo_2O_4$ above 0 eV to the energy level of 0 eV (vs NHE) fulfilling the electron accepting need for HER driven by the electric potential.

Similarly, the holes would transfer from the lowered Femi level that contains lots of TDOS marked by black arrows below -1.23 eV to the energy level where the OER occurred. After nitrogenation for $NiCo_2O_4$, there established an internal electric field within the heterojunction structure at interface between Ni_3N/CoN and $NiCo_2O_4$. This Interface electric field effect at the interface of Ni_3N/CoN and $NiCo_2O_4$ will benefit for electrons transfer during the electrochemical reactions. As can be seen from the Figure 7, there would establish a new Femi level at the same horizon across the TDOS of the three compounds after nitrogenation. According to the principle that the two energy levels gets closer, the electrons transferred more easily, electrons would migrate from the Femi level of the NiCo-nitrides/$NiCo_2O_4$ dragged at the position that is close to the 0 eV and contains lots of TDOS marked by red arrows to the energy level of 0 eV during the HER process. Clearly, the CoN acts as the main contributor to facilitate the HER. In OER process, the positions that is close to the -1.23 eV and contains lots of TDOS marked by red arrows could be found in TODS of Ni_3N and secondarily CoN. Thus, Ni_3N acts as the main contributor to facilitate OER. Notably, we investigated the function of the Ni_3N and CoN derived from nitrogenation for $NiCo_2O_4$ by analyzing relationship between the electrons transferring and the Femi level changes comparing oxides with oxides/nitrides. The above analysis is shown to be in keeping with the experimental observations regarding the intrinsic electrocatalytic activity and the electrochemical reaction mechanism.

References

[1] G. Kresse, J. Hafner, Physical Review B (Condensed Matter) **1993**, 47, 558; G. Kresse, J. Hafner, Physical Review B (Condensed Matter) **1993**, 48, 13115; G. Kress, J. Furthmuller, Comput. Mater. Sci **1996**, 6, 15.

[2] G. Kresse, J. Furthmuller, Physical Review B (Condensed Matter) **1996**, 54, 11169; G. Kresse, D. Joubert, Physical Review B **1999**, 59, 1758.

[3] J. P. Perdew, K. Burke, M. Ernzerhof, Phys Rev Lett **1996**, 77, 3865.

[4] J. Y. Chen, X. Wu, A. Selloni, Physical Review B **2011**, 83, 245204; V. I. Anisimov, J. Zaanen, O. K. Andersen, Physical Review B **1991**, 44, 943.

[5] H. J. Monkhorst, J. D. Pack, Physical Review B (Solid State) **1976**, 13, 5188.

[6] X. Y. Yu, Y. Feng, Y. Jeon, B. Guan, X. W. Lou, U. Paik, Adv Mater **2016**, 28, 9006.
